# Disrupted-in-Schizophrenia (DISC1) Functions Presynaptically at Glutamatergic Synapses

**DOI:** 10.1371/journal.pone.0034053

**Published:** 2012-03-30

**Authors:** Brady J. Maher, Joseph J. LoTurco

**Affiliations:** 1 Department of Physiology and Neurobiology, University of Connecticut, Storrs, Connecticut, United States of America; 2 Lieber Institute for Brain Development, Johns Hopkins Medical Campus, Baltimore, Maryland, United States of America; The Research Center of Neurobiology-Neurophysiology of Marseille, France

## Abstract

The pathophysiology of schizophrenia is believed to involve defects in synaptic transmission, and the function of many schizophrenia-associated genes, including DISC1, have been linked to synaptic function at glutamatergic synapses. Here we develop a rodent model via *in utero* electroporation to assay the presynaptic function of DISC1 at glutamatergic synapses. We used a combination of mosaic transgene expression, RNAi knockdown and optogenetics to restrict both genetic manipulation and synaptic stimulation of glutamatergic neurons presynaptic to other layer 2/3 neocortical pyramidal neurons that were then targeted for whole-cell patch-clamp recording. We show that expression of the DISC1 c-terminal truncation variant that is associated with Schizophrenia alters the frequency of mEPSCs and the kinetics of evoked glutamate release. In addition, we show that expression level of DISC1 is correlated with the probability of glutamate release such that increased DISC1 expression results in paired-pulse depression and RNAi knockdown of DISC1 produces paired-pulse facilitation. Overall, our results support a direct presynaptic function for the schizophrenia-associated gene, DISC1.

## Introduction

DISC1 was identified as a schizophrenia susceptibility gene because a chromosomal translocation that results in a c-terminal truncation of the DISC1 gene was found to co-segregate with major mental illness in an extended Scottish pedigree [1], [2]. Studies have shown that DISC1 is a scaffolding protein with a variety of functions during all aspects of neural development [3], [4]. RNAi knockdown of DISC1 during early neocortical and hippocampal development resulted in several phenotypes including the disruption of neurogenesis, migration, altered dendritic arborization, and the density of spines [5], [6], [7], [8], [9]. Transgenic animals expressing a truncated version of DISC1 (DISC1ΔC) under control of the CaMKII promoter were shown to display abnormal behavioral phenotypes, enlarged ventricles, decreased levels of cortical dopamine, fewer parvalbumin-positive neurons and altered spine density [10], [11]. These studies provide strong evidence for DISC1 having important roles in postsynaptic physiology and structure, however evidence also exists that suggest DISC1 has important presynaptic functions.

DISC1 immunoreactivity is observed at the ultrastructural level in presynaptic terminals [12], [13]. Both acute RNAi knockdown and a knockin mouse that creates a truncating lesion in DISC1 resulted in altered axonal targeting of mossy fibers [14], [15]. The knockin mouse also produced changes in short-term plasticity at the mossy fiber/CA3 synapse [15]. Moreover, RNAi knockdown of DISC1 disrupts the transport of synaptic vesicles and mitochondria [16], [17], two cellular organelles important for synaptic transmission [18]. Here we use optogenetics and whole-cell electrophysiology to specifically test for a presynaptic function of DISC1 in cortical layer 2/3 pyramidal neurons. We show expression of DISC1ΔC enhances mEPSC frequency and alters the kinetics of the evoked glutamate transient. In addition, we show the expression level of DISC1 in presynaptic neurons regulates the probability of glutamate release. Overall, our data provide several lines of evidence that suggest DISC1 has direct functions in presynaptic transmission.

## Materials and Methods

### Ethics Statement

All studies were conducted in accordance with protocols that were approved by the University of Connecticut Institutional Animal Care and Use Committee (IACUC; Assurance No. A09-025, 2/2011). The facilities at the University of Connecticut are accredited by the Association for the Assessment and Accreditation of Laboratory Animal Care (AAALAC).

### *In utero* electroporation and inducible plasmid expression

*In utero* electroporation was performed on Wistar rats as previously described [19]. For inducible expression we used a 4-OHT-activatible form of Cre recombinase (pCAG-ER^T2^-CreER^T2^, 1.5 ug/µL) and Cre-dependent inducible expression vectors (pCALNL-dsRed, pCALNL-GFP, pCALNL-wtDISC1, and pCALNL-DISC1ΔC, 1.5 µg/µL; a gift from T. Matsuda and C. Cepko, Harvard Medical School, Boston). Both DISC1 and DISC1ΔC constructs were gifts from A. Kamiya and A. Sawa, John Hopkins School of Medicine, Balitmore, MD and were subcloned into the pCALNL plasmid for this study. The D1 shRNA (pUEG-D1) was a gift from H. Song, John Hopkins School of Medicine, Baltimore, MD. Channelrhodophsin plasmid (pcDNA3.1hChR2-EYFP) was a gift from K Diesseroth, Stanford University, Stanford, CA, and was subcloned into the pCAG plasmid for this study.

**Figure 1:**
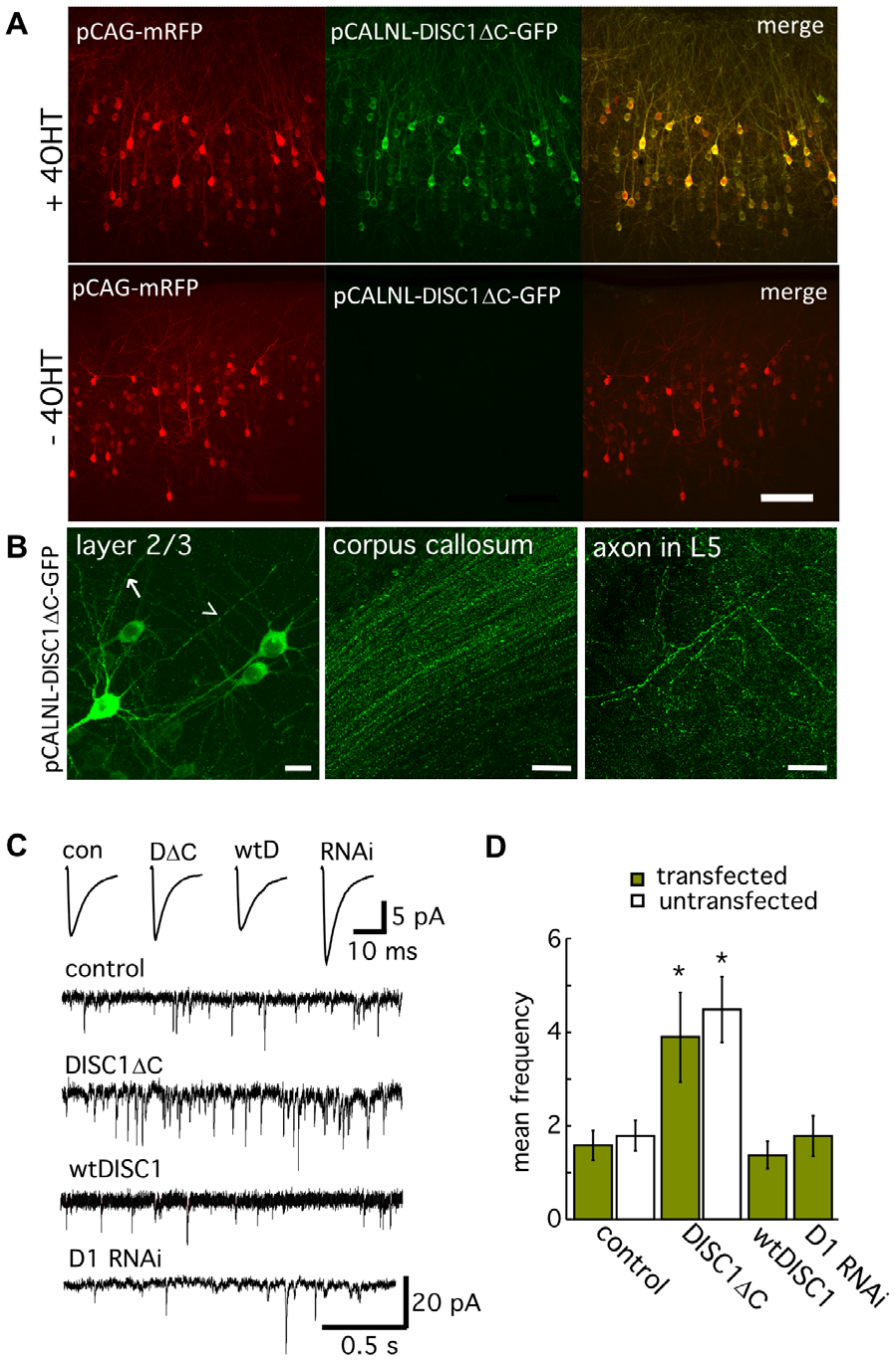
Postnatal expression of DISC1ΔC enhances the frequency of mEPSCs. A) Expression of DISC1ΔC is induced by postnatal administration of 4-OHT as seen by expression of GFP fused to DISC1ΔC in this P28 brain slice. No GFP expression is observed in vehicle treated animals (- 4-OHT). Scale bar equals 100 µm. B) DISC1ΔC-GFP is localized to the soma, dendrite (arrow), and axons (arrowhead) of layer 2/3 pyramidal cells. Expression is also observed in axon terminal field of layer 5 and axon tracts of the corpus callosum. Scale bar equals 20 µm. C) Example mEPSC sweeps from transfected layer 2/3 neurons expressing either GFP (control), DISC1ΔC, wtDISC1 or D1 RNAi. Traces shown above are the average of all the captured mEPSC from every recording for each condition. D) Summary graph showing the frequency of mEPSCs are nearly doubled by DISC1ΔC expression. This increase in synaptic activity was present to the same extent in both transfected cells expressing DISC1ΔC and neighboring non-transfected cells. All recordings performed in the presence of gabazine (5 µM) and TTX (1 µM).

**Figure 2:**
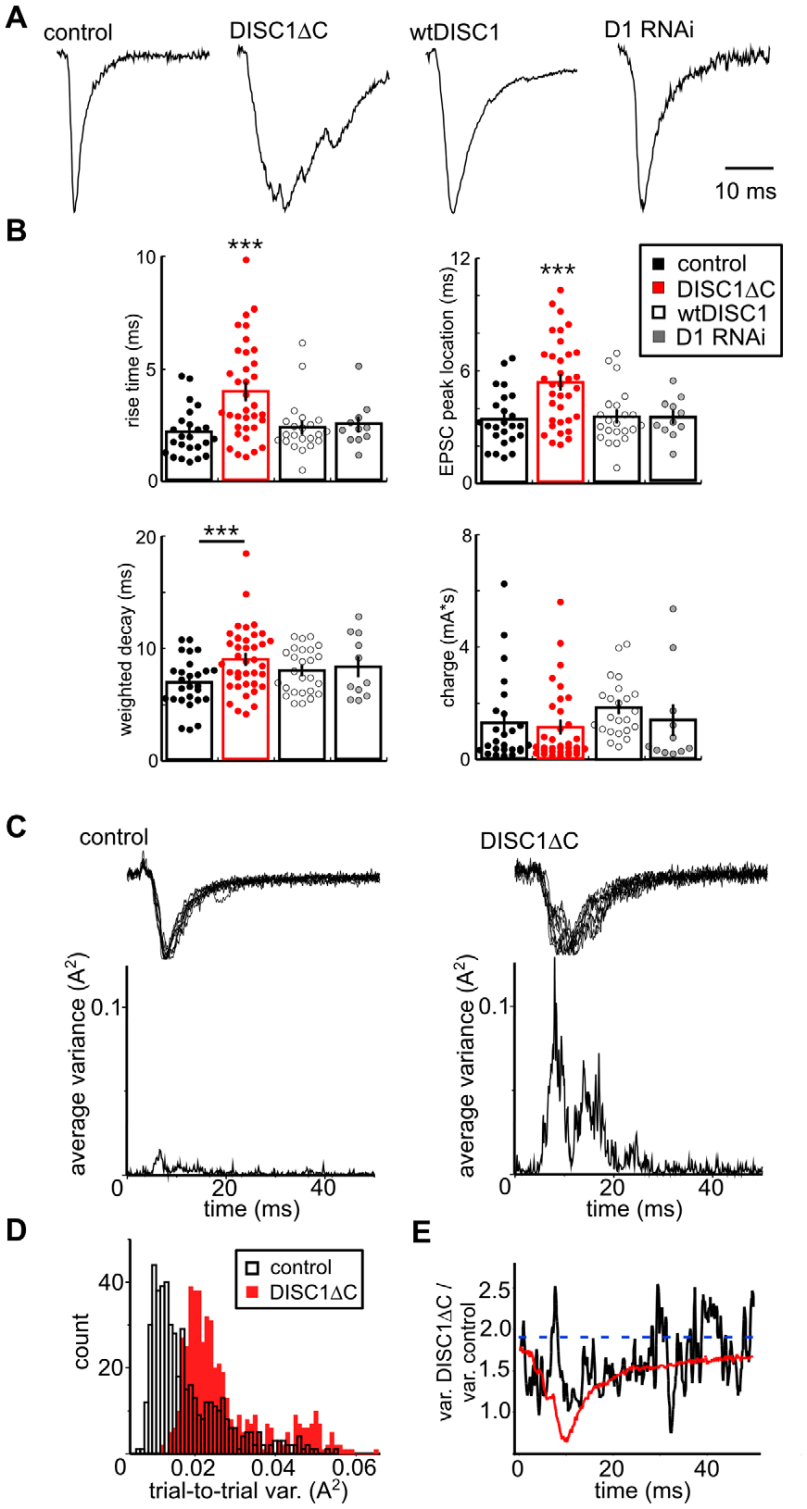
Presynaptic expression of DISC1ΔC alters the kinetics of evoked glutamate release and inhibits synchronous glutamate release. A) Normalized EPSCs recorded from an untransfected neuron and evoked from presynaptic terminals expressing ChR2 and either dsRed (control), DISC1ΔC, wtDISC1 or D1 RNAi. B) Summary graphs showing the mean ± SEM of four different kinetic measures of EPSCs evoked from presynaptic terminals expressing ChR2 and either dsRed (control), DISC1ΔC, wtDISC1 or D1 RNAi. C) Two representative recordings from a neuron in control or DISC1ΔC conditions showing 10 consecutive traces that are normalized and overlaid (top traces). The average trial-to-trial variance for the traces above is displayed over the duration of the waveform (bottom trace). D) A histogram depicting the average trial-to-trial variance for all neurons in each condition (bin = 0.001 A^2^). E) The ratio of the average trial-to-trial variance for each condition (black trace) is displayed over the duration of the EPSC waveform. The dotted blue line indicates the threshold for statistical significance (p<0.05; F-test). A representative DISC1ΔC EPSC (red trace) is overlaid to emphasize the aspects of the EPSC waveform that produce the most variance.

**Figure 3:**
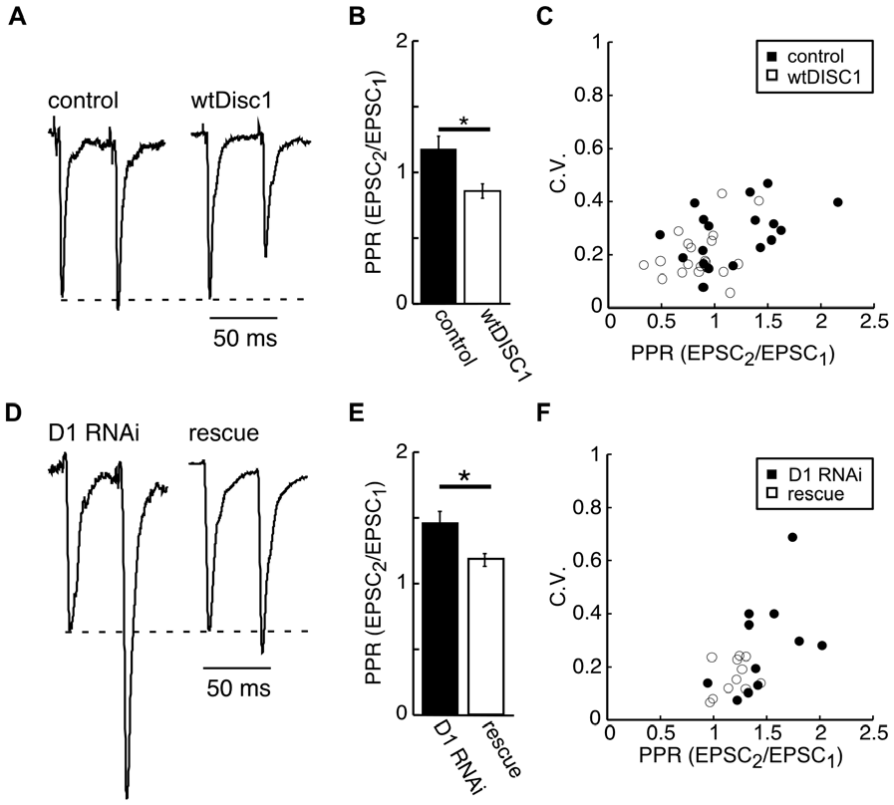
Presynaptic transmitter release is regulated by the expression levels of DISC1. A) Representative EPSCs recorded from an untransfected neuron and evoked from presynaptic neurons expressing ChR2 and either dsRed (control) or wtDISC1. B) Summary graph showing the mean±SEM PPR recorded from control and wtDISC1 conditions. C) Summary plot comparing the CV values vs PPR for each condition. D) Representative EPSCs evoked from neurons expressing DISC1 RNAi (D1 RNAi) and D1 RNAi+DISC1-GFP (rescue). E) Summary graph showing the mean±SEM PPR recorded from D1 RNAi and rescue conditions. F) Summary plot comparing CV values vs PPR for each condition. All EPSC traces are normalized to the first EPSC peak amplitude.

In all experiments, two conditions were created within the same dam. Visible expression of dsRed and/or GFP indicated reliable Cre-mediated recombination, marked transfected neurons and identified the experimental condition. For experiments in Figure 1 C–D, S1 and S2 embyros were injected with a combination of three plasmids, pCAG-ER^T2^-CreER^T2^, pCALNL-dsRed, and either pCALNL-GFP (control), pCALNL-wtDISC1, pCALNL-DISC1ΔC or pUEG-D1. For experiments using light-activated synaptic transmission (Figures 2, 3, S4, and S5), embyros were injected with pCAG-ER^T2^-CreER^T2^, pCAG-ChR2-venus, and either pCALNL-dsRed (control), pCALNL-wtDISC1, or pCALNL-DISC1ΔC. For D1 RNAi and rescue experiments embryos were injected with pCAG-ChR2-venus, pUEG-D1, and either pCAG-mRFP (D1 RNAi) or pCAG-wtDISC1-GFP.

The pCALNL-DISC1ΔC-GFP and pCAG-wtDISC1-GFP was made by fusing eGFP to the C-terminus. DISC1ΔC-GFP was only used in Figure 1A–B to demonstrate protein localization and wtDISC1-GFP was only used for RNAi rescue in Figure 3 D–F, all other experiments lacked the GFP fusion.

### RNAi validation

The D1 RNAi was previously shown to knockdown DISC1 in several mouse lines [6], [14], [20]. The D1 hairpin target sequence matches exactly to both mouse and rat DISC1 sequences (GGCTACATGAGAAGCACAG; nucleotide position 17701097–17701115; accession number NW_047536). We validated knockdown of rat DISC1 transcripts by co-transfecting pUEG-D1 or pUEG (control) and a DISC1 expression construct cloned from rat brain (pCAG-rDISC1-flag). pUEG-D1 produced a significant knockdown of rDISC1-flag protein compared to control (56.8±0.1% (n = 4) p = 0.013). Protein expression was quantified using LI-COR Odyssey in-cell western assay.

### 4-OHT administration

4-OHT administration was performed as described by Manent et al., 2009. Briefly, 4-hydroxytamoxifen (4-OHT; Sigma) was dissolved in 95% ethanol at a concentration of 20 mg/ml and diluted in 9 volumes of corn oil. Diluted 4-OHT (2 mg/100 g body weight) was administered to the animals via i.p. injection on P5 and P7. Vehicle-treated animals were injected with the same solution without 4-OHT.

### Histological procedures and microscopy

Animals (p28) were transcardially perfused under deep anesthesia with 4% paraformaldehyde in PBS. Brains were removed and post-fixed 24 hours in the same fixative solution, prior to coronal sectioning with a vibratome (Leica, Nussloch, Germany) Brain sections were processed for immunohistochemistry as floating sections. Primary antibody was goat-anti-GFP (1∶1000, Molecular Probes) and secondary antibody was rabbit-anti-goat conjugated with Alexa 488 (1∶200, Molecular Probes). Photomicrographs were taken with a Leica TCS SP2 confocal microscope (Nussloch, Germany) and Zeiss Axio Imager 2 with Zeiss ApoTome module. For spine counting, the primary basal dendrites of layer 2/3 pyramidal cells from at least three animals in each condition were imaged. All counting was done blind using Neurolucida (MBF Bioscience, Williston, VT) for analysis. For axon arborization measurements were performed as previously described [21]. Except, to compensate for variability in the efficacy of labeling callosal axons with GFP, the densiometric line scans were normalized by the average GFP intensity measured just above the white matter tract below the area of interest. To analyze the number of presynaptic active zones per length of axon we co-transfected layer 2/3 neurons with synpatophysin-RFP (pCAG-syp-RFP), pCAG-ER^T2^-CreER^T2^, pCALNL-GFP (control) or pCALNL-GFP plus pCALNL-DISC1ΔC. pCAG-syp-RFP was subcloned from pTRE-Bi-SG-T (Addgene plasmid 26084 [22]), fused with RFP and placed behind the pCAG promoter. NIH Image J Software was used to measure the length of contralateral axons and syp-RFP positive puncta were manually counted. All counts were done blind and at least three animals per condition were used.

### Preparation of acute brain slices

Acute brain slices were performed as previously described [23]. Briefly, P28–40 rats were deeply anesthetized with isoflurane and transcardially perfused with ice-cold oxygenated (95% O2 and 5% CO2) dissecting buffer containing (in mM): 83 NaCl, 2.5 KCl, 1 NaH2PO4, 26.2 NaHCO3, 22 glucose, 72 sucrose, 0.5 CaCl2, and 3.3 MgCl2. The rat was decapitated and the brain was rapidly removed and immersed in ice-cold oxygenated dissection buffer. Coronal slices (400 µm) were cut using a vibratome (VT1200S, Leica), incubated in dissection buffer for 30–45 min at 34°C, and then stored at room temperature. Slices were visualized using IR differential interference microscopy (DIC) (E600FN, Nikon) and a CCD camera (QICAM, QImaging). Individual layer 2/3 pyramidal cells expressing GFP, dsRed and/or ChR2-venus were visualized with epifluourescent illumination and a 40× Nikon Fluor water immersion (0.8 numerical aperture) objective.

### Electrophysiology

For all experiments, artificial cerebrospinal fluid (ACSF) was oxygenated (95% 02 and 5% CO2) and contained (in mM): 125 NaCl, 25 NaHCO3, 1.25 NaH2PO4, 3 KCl, 25 dextrose, 1 MgCl2, and 2 CaCl2, pH 7.3. Patch pipettes were fabricated from borosilicate glass (N51A, King Precision Glass, Inc.) to a resistance of 2–5 MΩ. For current-clamp experiments and mEPSC measurements pipettes were filled with (in mM): 125 potassium gluconate, 10 HEPES, 4 Mg-ATP, 0.3 Na-GTP, 0.1 EGTA, 10 phosophocreatine, 0.05% biocytin, adjusted to pH 7.3 with KOH. For EPSC measurements pipettes contained (in mM): 110 CsMeSO4, 10 CsCl, 10 HEPES, 10 Cs4-BAPTA, 5 QX314 Br, 0.1 spermine, 4 Mg-ATP, 0.4 Na-ATP, 10 phosophocreatine, 0.05% biocytin, adjusted to pH 7.3 with KOH. For all experiments GABAa receptors were blocked with SR-95531 (Gabazine, 5 µM, Ascent Scientific). For some experiments, synaptic currents were blocked with DL-2-amino-5-phosphopentanoic acid (D,L-AP5, 100 µM, Ascent Scientific), 2,3, dioxo-6-nitro-1,2,3,4, tetrahydrobenzo-quinoxaline-7-sulfonamide (NBQX, 10 µM), and tetrodotoxin (TTX, 1 µM; Ascent Scientific). Current signals recorded with a Multiclamp 700A amplifier (Molecular Devices) were filtered at 2 kHz using a built in Bessel filter and digitized at 10 kHz. Data were acquired using Axograph. Data acquisition was terminated when series resistance was >15 MΩ. For voltage clamp recordings, pyramidal cells were held at −70 mV.

### Light-activated synaptic transmission

ChR2-venus was activated by 2 ms pulses of blue light (473 nM; ∼1 mW) from a 20 mW laser (Dragon Lasers, China) attached to a fiber optic cable. The end of the fiber optic cable was attached to a ceramic patch pipette holder and manipulator. The tip of the fiber optic cable was submerged into the bath above the brain slice. Light-evoked EPSC amplitudes were monitored as the fiber optic was moved until the maximum evoked amplitude was achieved. Gabazine (5 µM) was then washed into the bath to block feedforward inhibition and the underlying EPSC was revealed.

### Data Analysis and Statistics

We used Axograph on a Macintosh computer for analysis. For analysis of EPSC kinetics the rise time equals the duration of time between 10% and 90% of the maximum peak amplitude. EPSC peak location equals the duration of time between the EPSC onset (5% of the peak amplitude) and the maximum peak amplitude. Weighted decay equals the total charge from the peak of the response back to baseline divided by the peak amplitude. EPSC charge was measured for 50 ms following the EPSC onset. EPSC rise time and peak location was measured from normalized EPSCs that were the average of 10 consecutive sweeps. For all experiments, statistical significance was determined using standard t-tests, 1-way ANOVA with Student Newman-Keuls post-hoc test. All statistical significance is indicated on the figures with asterisks. Averaged data values are reported as mean ± SEM.

## Results

In order to determine whether DISC1 regulates synaptic transmission we used *in utero* electroporation to alter DISC1 expression in approximately 20% of neocortical layer 2/3 pyramidal neurons by conditionally expressing full-length DISC1 (wtDISC1), DISC1ΔC, or constitutive expression of an shRNA previously shown to create effective RNAi knockdown of DISC1 (D1 RNAi [6], [14], [20]; see methods). *In utero* electroporation produces high fidelity co-transfection of multiple plasmids and reliable inducible expression with no leaky expression in the absence of tamoxifen (Figure 1A [19]). Conditional expression of DISC1ΔC on P5 is after neurogenesis and neuronal migration is complete and therefore does not result in early developmental disruptions as previously reported for embryonic expression [5]. To identify the cellular localization of our inducible DISC1ΔC construct we fused it with GFP (pCAG-DISC1ΔC-GFP) and observed expression in the soma, dendrites, axons (Figure 1B), and axon terminals of layer 2/3 pyramidal neurons (Figure 1C). This distribution throughout the neuron is consistent with previous studies showing nearly ubiquitous distribution of DISC1 [12].

We next used whole-cell patch clamp recording to characterize effects of DISC1ΔC expression on the electrophysiology of neocortical pyramidal neurons. We found no significant differences in the intrinsic membrane properties, including resting membrane potential, input resistance and spike firing rates in neurons expressing DISC1ΔC, wtDISC1, D1 RNAi or GFP (Figure S1). However, the frequency of miniature excitatory synaptic currents (mEPSCs) mediated by glutamatergic synaptic activity was nearly doubled by DISC1ΔC expression (Figure 1C,D; control transfected 1.58±0.31 (n = 9), control untransfected 1.79±0.32 (n = 5), DISC1ΔC transfected 3.90±0.96 (n = 10), DISC1ΔC untransfected 4.49±0.7 (n = 6), wtDISC1 transfected 1.37±0.29 (n = 7); ANOVA p<0.002). This increase in synaptic activity was present to the same extent in both transfected cells expressing DISC1ΔC and neighboring non-transfected cells and indicates presynaptic expression of DISC1ΔC is sufficient to explain the increase in mEPSC frequency. DISC1ΔC expression had no significant on mEPSC amplitudes, rise times, or decays, compared to wtDISC1 or GFP controls (but see D1 RNAi; Figure S2A–C). Furthermore, we observed no significant difference in the density of spines, complexity of contralateral axonal projections, or the density of presynaptic terminals as visualized with a synaptophysin-RFP (syp-RFP) fusion protein (Figure S2D,E). Together, these results suggest the DISC1ΔC-dependent enhancement of mEPSC frequency is not due to increases in synapse number, but rather reflects an alteration in presynaptic function.

To directly test the presynaptic function of DISC1 we combined our genetic manipulations with co-expression of channelrhopsin (ChR2). We then stimulated this transfected population of neurons with a laser pulse and recorded evoked excitatory postsynaptic currents (EPSCs) from untransfected layer 2/3 neurons (Figure S4B). Any altered synaptic transmission recorded in untransfected neurons would be due to manipulation of DISC1 in the photoactivated presynaptic neurons. In all conditions, laser stimulation reliably evoked single EPSCs that were completely blocked by TTX or NBQX (Figure S4B1), confirming that the light-stimulated population of neurons was indeed presynaptic to the untransfected recorded cells.

We next assayed how expression of our DISC1 constructs would affect the light-evoked glutamate release by monitoring EPSC kinetics. Presynaptic stimulation of neurons expressing DISC1ΔC consistently produced unusual multiphasic EPSCs that exhibited significantly slower rise-times and significantly delayed EPSC peaks compared to GFP controls, wtDISC1 and D1 RNAi (Figure 2A,B; rise time control 2.17±0.22 ms (n = 24) vs. DISC1ΔC 3.98±0.36 ms (n = 35); p<0.001; DISC1ΔC vs. wtDISC1 2.40±0.28 ms (n = 23); p<0.002; DISC1ΔC vs. D1 RNAi 2.52±0.31 ms (n = 11); p<0.01; peak location control 3.40±0.30 ms (n = 24) vs. DISC1ΔC 5.35±0.38 ms (n = 35); p<0.001; DISC1ΔC vs. wtDISC1 3.54±0.35 ms (n = 23); p<0.001; DISC1ΔC vs. D1 RNAi 3.52±0.32 ms (n = 11); p<0.02). In addition, presynaptic expression of DISC1ΔC significantly prolonged EPSC decay times compared to controls, but not compared to wtDISC1 or D1 RNAi (Figure 2A,B; control 6.9±0.5 ms (n = 24) vs. DISC1ΔC 8.9±0.5 ms (n = 34); p<0.01, wtDISC1 8.1±0.4 ms (n = 23); p = 0.27, D1 RNAi 8.34±0.83 ms (n = 11); p = 0.45). We did not observe a significant difference in the total EPSC charge between conditions (Figure 2B; ANOVA p = 0.24) suggesting that the total number of vesicles released is unchanged by DISC1ΔC expression.

DISC1ΔC-dependent slowing of glutamate release was not due to an effect on action potential generation. Using cell attached recordings from either DISC1ΔC or GFP transfected neurons, we did not observe a difference in the trial-to-trial temporal jitter of action potentials that were generated by light activation of ChR2. Recordings from GFP (n = 9) or DISC1ΔC (n = 8) transfected neurons showed less than a 50 µs variation in the to time to action potential peak from trial-to-trial. This temporal variation is significantly less than what is observed for the DISC1ΔC–dependent slowing of EPSC kinetics (approx. 2 ms).

One possibility for slowed kinetics observed in averaged synaptic responses, as measured above, is that presynaptic expression of DISC1ΔC may increase trial-to-trial variability in the EPSC waveform. To determine and temporally map the possible change in variability in response, we normalized 10 consecutive responses to their peak amplitude for each recording from control and DISC1ΔC transfection conditions and computed the average trial-to-trial variance over the duration of the EPSC waveform (Figure 2C). The averaged time-resolved variances for responses for all recordings show a significant increase in the trial-to-trial variance across time for DISC1ΔC (n = 34) compared to control (Figure 2d; (n = 24)). Moreover, the difference in variance is maximally different during the rise-time and decay of the EPSC (Figure 2E) suggesting further a desynchronization in vesicle release across the entire EPSC waveform. These results together indicate that presynaptic expression of DISC1ΔC inhibits the synchronous nature of vesicle release, an effect distinct from the effects of DISC1 knockdown or DISC1 overexpression and confirms previous reports that DISC1ΔC acts as a dominant negative construct [5].

The specific presynaptic mechanisms that underlie the effects of DISC1ΔC are unknown at this time, however they are not related to measurable changes in intrinsic membrane properties, presynaptic excitability (Figure S1) or axonal structure (Figure S3). Many central synapses display asynchronous release or delayed release that is observed during periods of high frequency stimulation, and this property of asynchronous release is believed to be separate from synchronous release [24]. DISC1ΔC–dependent slowing of EPSC kinetics observed here does not appear to be associated with changes in this type of asynchronous release, as we did not observe a slow build-up of charge during high frequency stimulation nor did we observe an increase in spontaneous EPSC (sEPSC) frequency following high frequency stimulation (Figure S5). This suggests that the DISC1ΔC effect on desynchronizing release is something distinct from asynchronous release.

We next asked if the expression level of DISC1 could regulate the probability of release by measuring paired-pulse ratios (PPR) and the coefficient of variation (CV). Light-evoked release from control neurons expressing GFP at 50 ms interval on average resulted in a slight paired-pulse facilitation (PPF; control, Figure 3; PPR = 1.17±0.08, n = 18). In contrast, light stimulation of cells expressing wtDISC1 consistently resulted in paired-pulse depression (PPD; wtDISC1, Figure 1b–d; PPR = 0.86±0.07, n = 21; p<0.02 vs. control). Paired-pulse depression indicates that the probability of transmitter release is enhanced by overexpression of wtDISC1, and this is supported by a correspondingly lower coefficient of variation (CV) in synaptic events produced by stimulating wtDISC1 expressing cells compared to GFP expressing controls (wtDISC1 0.20±0.02, n = 21 vs. control 0.28±0.03, n = 18; p<0.02). Conversely, knockdown of DISC1 by D1 RNAi resulted in significantly increased PPF (Figure 1e–g; PPR = 1.46±0.09, n = 11; p<0.02 vs. rescue) and increased CV values compared to a rescue control in which wtDisc1 was re-expressed (D1 0.28±0.05, n = 11 vs rescue 0.17±0.02, n = 11; p<0.04). The rescue of the effect of RNAi in both paired-pulse facilitation and CV measures rules out the possibility of off-target RNAi effects as being responsible for the changes in presynaptic release probability (Figure 1e–g; PPR = 1.18±0.05, n = 11; p = 0.90 vs control). The abnormal EPSC kinetics and increased trial-to-trial variability observed when DISC1ΔC was expressed precluded our ability to use peak amplitudes to analyze the probability of release from this condition. Together, these results indicate the level of DISC1 expression in presynaptic neurons regulates the probability of transmitter release such that overexpression of DISC1 enhances release probability and decreased DISC1 expression lowers the release probability.

## Discussion

We provide several lines of evidence showing DISC1 regulates glutamate release from presynaptic terminals. We show expression of DISC1ΔC enhances the frequency of mEPSCs and disrupts the synchronous nature of evoked glutamate release. Furthermore, we show the expression level of DISC1 in presynaptic neurons is correlated with the probability of glutamate release. Our results suggest RNAi knockdown of DISC1 produces different effects from those seen with expression of DISC1ΔC, and indicates DISC1ΔC acts as a dominant negative as suggested by others [5], [10].

Understanding the function of DISC1ΔC is relevant to schizophrenia not only because of the chromosomal translocation segregates with mental illness in the Scottish pedigree [1], [2], but also because several alternative splice variants of DISC1 were found to have higher expression in patients with schizophrenia [25]. Expression of DISC1ΔC under the control of the CaMKII promoter in a transgenic mouse line resulted in several phenotypes related to schizophrenia including enlarged lateral ventricles, reduction in parvalbumin immunoreactivity, reduced cortical dopamine levels and behavioral abnormalities [10]. Another mouse model that more closely models the human translocation by introducing a truncating lesion in the endogenous murine *Disc1* ortholog showed several presynaptic phenotypes including, abnormal axonal targeting in hippocampus, altered short-term synaptic plasticity, decreased volume of synaptic vesicles, and elevated cAMP levels [15]. DISC1ΔC may alter DISC1 function through its interaction with full-length DISC1. In cell models, truncated DISC1 was shown to form dimers with wild-type DISC1 that resulted in abnormal microtubule dynamics and defects in neuronal migration [5]. We show that postnatal expression of DISC1ΔC results in an enhancement of mEPSC frequency in both transfected and neighboring untransfected neurons, suggesting either an enhancement in structural synaptic connectivity or an alteration in spontaneous vesicle fusion, or both. We therefore assessed whether DISC1ΔC changed morphological measures of connectivity in cortex. We compared dendritic spine densitities, axonal arborization, and the density of presynaptic active zones labelled by synaptophysin-mRFP fusion (syp-RFP) between control and DISC1ΔC expressing neurons, and found no significant evidence for DISC1ΔC altering any of these morphological measures of connectivity (Figure S5). This lack of effect on connectivity mirrors those obtained from a transgenic mouse model in which expression of truncated DISC1 was induced postnatally and no effect on spine density was observed [11]. Therefore these results suggest that the enhancement of mEPSC frequency by DISC1ΔC, are due to functional changes in transmitter release that are largely independent of changes in axonal sprouting or changes in spine number.

To further investigate the presynaptic function of DISC1 we utilized optogenetics to specifically stimulate presynaptic neurons expressing our DISC1 constructs. This analysis produced several distinct presynaptic phenotypes including effects on the kinetics of evoked glutamate release and the probability of glutamate release. Similar to the effects on mEPSC frequency, the kinetics of glutamate release was only altered by expression of DISC1ΔC. We observed that expression of DISC1ΔC increased the trial-to-trial variance over the duration of the EPSC waveform. One explanation for this result is that DISC1ΔC disrupts synchronous vesicle fusion normally observed at these synapses. The molecular mechanism responsible for this effect is currently unknown. However, an intriguing candidate mechanism involves the major Ca^2+^-sensor for vesicle fusion, synpatotagmin.

DISC1 interacts indirectly with synaptotagmin through an interaction with FEZ-1, and expression of DISC1ΔC was shown to attenuate synaptic vesicle transport in primary cortical neuronal cultures [16]. Genetic deletion of synaptotagmin results in a complete loss of synchronous release, dramatically enhances spontaneous vesicle fusion, and has very little effect on asynchronous release [24], [26], [27], [28]. These synaptotagmin-dependent effects on synchronous release are strikingly similar to our results observed by overexpression of DISC1ΔC, whereby DISC1ΔC appears to inhibit synchronous release in a dominant negative manner while also enhancing the frequency of spontaneous vesicle fusion. Future experiments directed at FEZ-1 expression may provide insight into this potential mechanism.

Our results also indicate DISC1 expression can regulate the probability of glutamate release, whereby overexpression of DISC1 results in paired-pulse depression and an increase in CV. Conversely, RNAi knockdown of DISC1 produces paired-pulse facilitation and a decrease in CV. These results further indicate a presynaptic function for DISC1, however the mechanism associated with this phenotype is not apparent. One potential mechanism involves DISC1 regulation of synaptic vesicle trafficking [16]. Alternatively, the regulation of mitrochondria trafficking by DISC1 may be important, as defects in the trafficking of mitochondria are known to alter several aspects of synaptic transmission including short-term plasticity [17], [18], [29], [30].

Overall, our results provide the strong evidence for presynaptic effects of DISC1 at glutamatergic synapses in the neocortex. The level of DISC1 expression appears to regulate the probability of release and therefore may function to control the reliability of glutamate release. In contrast, expression of DISC1ΔC appears to inhibit synchronous glutamate release and may consequently affect the timing of synaptic transmission through neocortical circuits.

## Supporting Information

Figure S1 **Analysis of membrane properties and neuronal excitability for neurons transfected with DISC1ΔC, wtDISC1, D1 RNAi or GFP.** A) Representative current-clamp recording showing the change in membrane potential to varying amounts of current injection (−200 pA to +300 pA). B) IV plot for all four conditions. C) Input/Output curve depicting the relationship between the amount of current injected and the number of action potentials generated (ANOVA p = 0.21). D) Group data showing the threshold for action potential generation (control −42.2±1.6 mV (n = 6); DISC1ΔC −40.7±0.4 mV (n = 6); wtDISC1 −43.6±1.6 mV (n = 8); D1 RNAi −40.8±1.42 mV (n = 13) ANOVA p = 0.53). E) Group data showing the average input resistance across experimental conditions (control 81.7±10.6 MΩ (n = 6); DISC1ΔC 78.7±18.3 MΩ (n = 6); wtDISC1 85.5±10.2 MΩ (n = 8); D1 RNAi 86.1±12.1 (n = 13); ANOVA p = 0.98). F) Group data showing the average resting membrane resistance for all four conditions (control −75.2±2.5 mV (n = 6); DISC1ΔC −72.2±0.9 mV (n = 6) p = 0.42 vs control; wtDISC1 −74.8±1.1 mV (n = 8) p = 0.86 vs control; D1 RNAi −70.2±1.2 mV (n = 13) p = 0.07 vs control, p = 0.04 vs wtDisc1). All data mean ± SEM; ANOVA with Student-Newman-Keuls Multiple Comparison post hoc test.
(TIF)

Figure S2 **Analysis of mEPSC amplitude and kinetics.** A) Summary graph showing knockdown of DISC1 significantly increases mEPSC amplitudes in neurons compared to transfected (green) or untransfected (open) neurons from brains expressing either dsRed (control), DISC1ΔC, or wtDISC1 (D1 RNAi transfected 14.45±0.8 pA; ANOVA p<0.001 vs. all groups (n = 8), control transfected 10.58±0.5 pA (n = 9), control untransfected 10.11±0.5 pA (n = 5), DISC1ΔC transfected 11.3±0.4 pA (n = 10), DISC1ΔC untransfected 11.1±0.2 pA (n = 6), wtDISC1 transfected 9.7±0.3 pA (n = 7), and suggests decreasing DISC1 expression may regulate the number of postsynaptic glutamate receptors. All recordings performed in the presence of gabazine (5 µM) and TTX (1 µM). B) Summary graph showing no significant difference in the mean ± SEM of the mEPSC rise time for each transfection condition (control transfected 0.65±0.05 (n = 9), control untransfected 0.66±0.04 (n = 5), DISC1ΔC transfected 0.56±0.02 (n = 10), DISC1ΔC untransfected 0.65±0.06 (n = 6), wtDISC1 transfected 0.56±0.01 (n = 7); ANOVA p = 0.20. C) Summary graph showing no significant difference in the mean ± SEM of the mEPSC weighted decay for each transfection (control transfected 4.35±0.50 (n = 9), control untransfected 4.23±0.24 (n = 5), DISC1ΔC transfected 3.33±0.24 (n = 10), DISC1ΔC untransfected 3.72±0.25 (n = 6), wtDISC1 transfected 3.45±0.12 (n = 7); ANOVA p = 0.13).
(TIF)

Figure S3 **Postnatal expression of DISC1ΔC does not alter pre- or postsynaptic structures.** A) Three representative images showing the basal dendrite of a layer 2/3 pyramidal neuron co-transfected with GFP and either dsRed (control), DISC1ΔC or wtDISC1. B) Summary graph showing no significant difference in the mean ± SEM of the number of spines per 1 µm of dendrite for each transfection condition (control 0.13±0.04 (n = 12), DISC1ΔC 0.16±0.05 (n = 10), wtDISC1 0.14±0.05 (n = 9); ANOVA p = 0.21). C) A representative coronal brain section showing the contralateral axons from neurons co-transfected with GFP and DISC1ΔC. Line scans were used to measure the average signal intensity across the entire cortex and were normalized by the signal intensity found just above corpus colossum. The line scans were binned and averaged within each condition D) Summary plots showing no significant difference in the amount of axonal arborization between control and DISC1ΔC conditions. E) For statistical analysis data each condition was binned by anatomical layer. No significant difference was observed within each layer between each condition (control (n = 7) vs. DISC1ΔC (n = 6); layer 2/3 1.48±0.10 vs. 1.46±0.06 p = 0.81; layer 4 1.17±0.05 vs. 1.09±0.08 p = 0.41; layer 5 1.19±0.03 vs. 1.12±0.02 p = 0.09; layer 6 1.04±0.02 vs. 0.94±0.05 p = 0.08) F) A representative image of contralateral axons co-expressing GFP, synaptophysin-RFP (syp-RFP) and DISC1ΔC. Arrowheads show syp-RFP positive puncta that colocalize with GFP positive axonal varicosities. G) Group data showing no significant difference in the number of syp-RFP puncta per length of axon (control (n = 17) vs. DISC1ΔC (n = 12); p = 0.34).
(TIF)

Figure S4 **Activation of ChR2 with a 2 ms pulse of blue light evokes glutamatergic synaptic transmission.** *In utero* electroporation on E15–E16 results in transfection of approximately 20% of layer 2/3 neurons in the targeted cortical area. Measuring light-activated synaptic transmission is amendable in this circuit because of the high level of recurrent connections between layer 2/3 neurons. A) Schematic depicting a recording in a layer 2/3 neuron transfected with ChR2 (green cell). (A1) A 2 ms pulse of blue light (473 nM; ∼1 mW) generates a single action potential in a control neuron that is blocked by TTX application. B) Schematic depicting a recording from an untransfected neuron (open cell) surrounded by layer 2/3 neurons transfected with ChR2. B1) A 2 ms pulse of blue light stimulates surrounding ChR2-positive neurons to fire action potentials and results in an EPSC in the untransfected neuron that is completely blocked by TTX (1 µM, 94.4±4.8% block of control response, n = 13, p<0.005) or the AMPA receptor antagonist NBQX (10 µM, 96.5±0.8% block of control response, n = 11, p<0.0005). All EPSC recordings performed in the presence of the GABAa antagonist (gabazine, 5 µM).
(TIF)

Figure S5 **Presynaptic expression of DISC1ΔC does not alter asynchronous release.** A) A representative train of EPSCs recorded from an untransfected neuron and evoked from presynaptic neurons expressing ChR2 and dsRed (control). Ten consecutive traces are overlaid. EPSCs were evoked with a train of 15 light pulses at 50 Hz. sEPSCs were collected 400 ms before and after the stimulus train (inset). B) A representative train of EPSCs recorded from an untransfected neuron and evoked from presynaptic neurons expressing ChR2 and DISC1ΔC. C) Summary data showing this stimulation protocol was effective in producing a significant asynchronous release for control recordings, as the frequency of sEPSCs is significantly enhanced following the stimulus train in control but not DISC1ΔC condition (control frequency before train 1.2±0.2 Hz vs. after train 2.6±0.6 Hz (n = 12); p<0.02 paired t-test; DISC1ΔC frequency before train 2.1±0.4 Hz vs. after train 2.6±0.4 Hz (n = 13); p = 0.2). However, the post stimulation/prior stimulation ratio of sEPSC frequency was not statistically different between control and DISC1ΔC terminals (control 2.12±0.36 (n = 12) vs. DISC1ΔC 1.48±0.19 (n = 13); p = 0.13), suggesting the DISC1ΔC–dependent slowing of EPSC kinetics is separate from asynchronous release.
(TIF)
