# Supplementary figures and images for: Disrupted-in-Schizophrenia (DISC1) Functions Presynaptically at Glutamatergic Synapses
Source: PLoS One. 2012 Mar 30;7(3):e34053. doi: 10.1371/journal.pone.0034053 (PMC3316587; doi:10.1371/journal.pone.0034053)

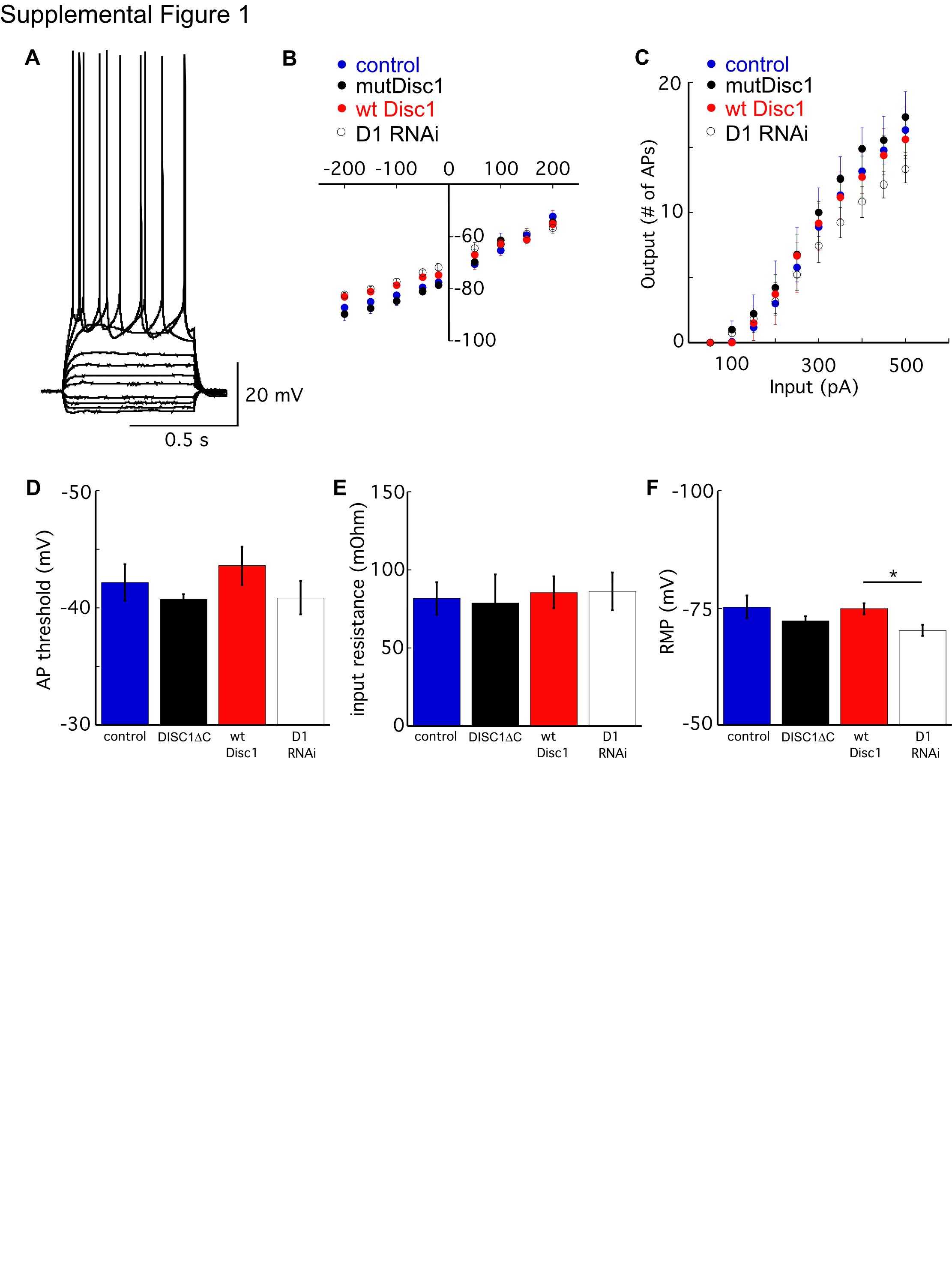

Supplement: Figure S1 — Analysis of membrane properties and neuronal excitability for neurons transfected with DISC1ΔC, wtDISC1, D1 RNAi or GFP. A) Representative current-clamp recording showing the change in membrane potential to varying amounts of current injection (−200 pA to +300 pA). B) IV plot for all four conditions. C) Input/Output curve depicting the relationship between the amount of current injected and the number of action potentials generated (ANOVA p = 0.21). D) Group data showing the threshold for action potential generation (control −42.2±1.6 mV (n = 6); DISC1ΔC −40.7±0.4 mV (n = 6); wtDISC1 −43.6±1.6 mV (n = 8); D1 RNAi −40.8±1.42 mV (n = 13) ANOVA p = 0.53). E) Group data showing the average input resistance across experimental conditions (control 81.7±10.6 MΩ (n = 6); DISC1ΔC 78.7±18.3 MΩ (n = 6); wtDISC1 85.5±10.2 MΩ (n = 8); D1 RNAi 86.1±12.1 (n = 13); ANOVA p = 0.98). F) Group data showing the average resting membrane resistance for all four conditions (control −75.2±2.5 mV (n = 6); DISC1ΔC −72.2±0.9 mV (n = 6) p = 0.42 vs control; wtDISC1 −74.8±1.1 mV (n = 8) p = 0.86 vs control; D1 RNAi −70.2±1.2 mV (n = 13) p = 0.07 vs control, p = 0.04 vs wtDisc1). All data mean ± SEM; ANOVA with Student-Newman-Keuls Multiple Comparison post hoc test. (TIF) [file pone.0034053.s001.tif]

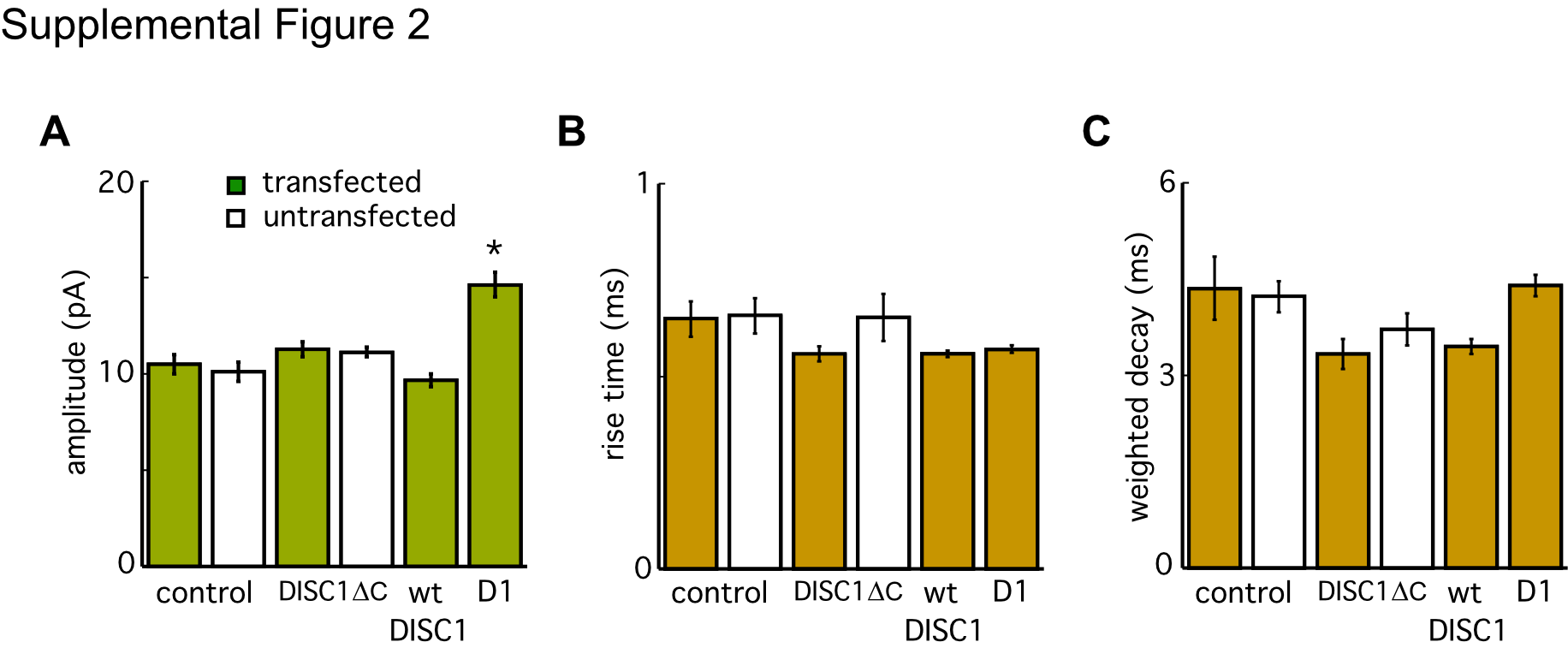

Supplement: Figure S2 — Analysis of mEPSC amplitude and kinetics. A) Summary graph showing knockdown of DISC1 significantly increases mEPSC amplitudes in neurons compared to transfected (green) or untransfected (open) neurons from brains expressing either dsRed (control), DISC1ΔC, or wtDISC1 (D1 RNAi transfected 14.45±0.8 pA; ANOVA p<0.001 vs. all groups (n = 8), control transfected 10.58±0.5 pA (n = 9), control untransfected 10.11±0.5 pA (n = 5), DISC1ΔC transfected 11.3±0.4 pA (n = 10), DISC1ΔC untransfected 11.1±0.2 pA (n = 6), wtDISC1 transfected 9.7±0.3 pA (n = 7), and suggests decreasing DISC1 expression may regulate the number of postsynaptic glutamate receptors. All recordings performed in the presence of gabazine (5 µM) and TTX (1 µM). B) Summary graph showing no significant difference in the mean ± SEM of the mEPSC rise time for each transfection condition (control transfected 0.65±0.05 (n = 9), control untransfected 0.66±0.04 (n = 5), DISC1ΔC transfected 0.56±0.02 (n = 10), DISC1ΔC untransfected 0.65±0.06 (n = 6), wtDISC1 transfected 0.56±0.01 (n = 7); ANOVA p = 0.20. C) Summary graph showing no significant difference in the mean ± SEM of the mEPSC weighted decay for each transfection (control transfected 4.35±0.50 (n = 9), control untransfected 4.23±0.24 (n = 5), DISC1ΔC transfected 3.33±0.24 (n = 10), DISC1ΔC untransfected 3.72±0.25 (n = 6), wtDISC1 transfected 3.45±0.12 (n = 7); ANOVA p = 0.13). (TIF) [file pone.0034053.s002.tif]

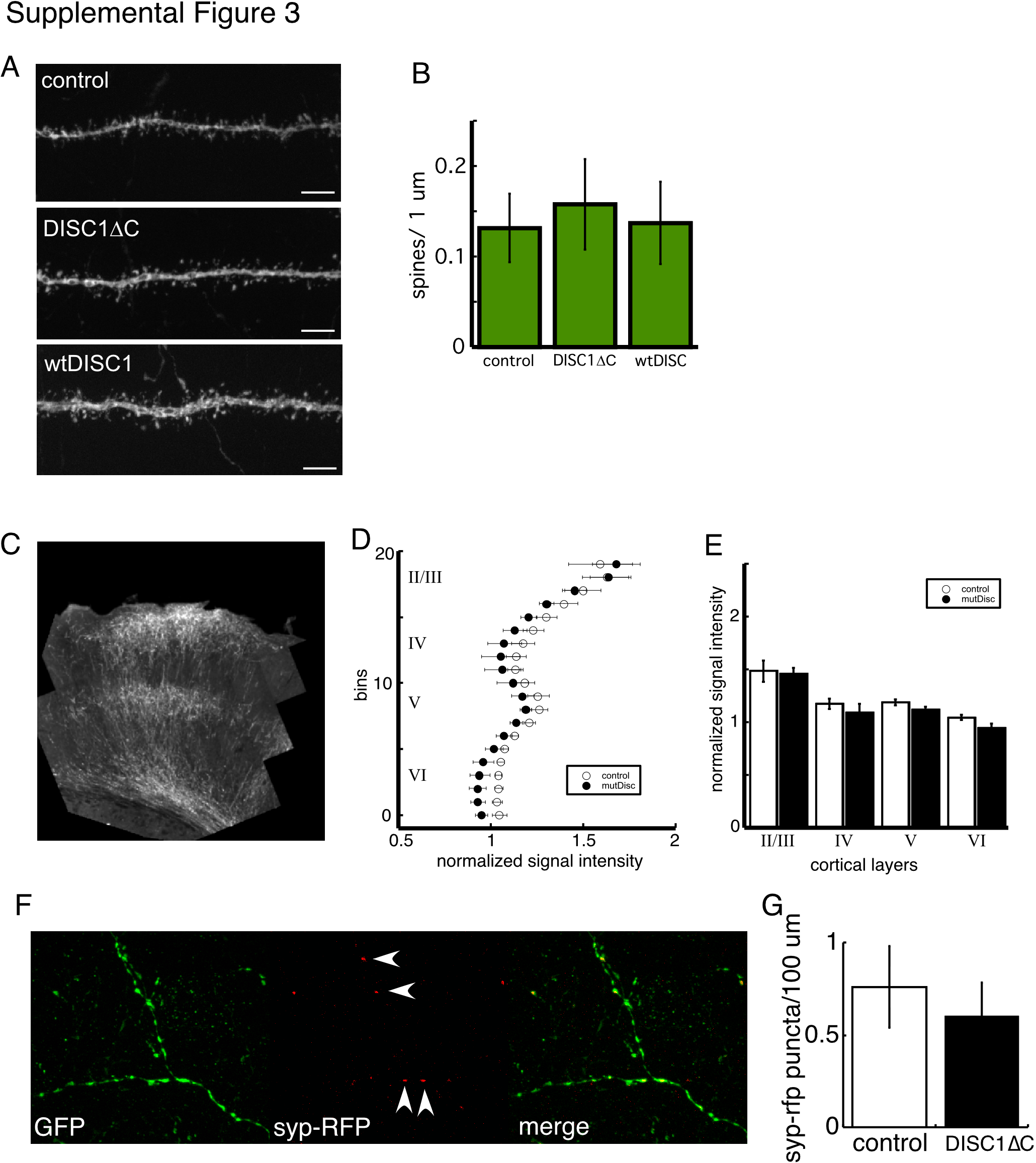

Supplement: Figure S3 — Postnatal expression of DISC1ΔC does not alter pre- or postsynaptic structures. A) Three representative images showing the basal dendrite of a layer 2/3 pyramidal neuron co-transfected with GFP and either dsRed (control), DISC1ΔC or wtDISC1. B) Summary graph showing no significant difference in the mean ± SEM of the number of spines per 1 µm of dendrite for each transfection condition (control 0.13±0.04 (n = 12), DISC1ΔC 0.16±0.05 (n = 10), wtDISC1 0.14±0.05 (n = 9); ANOVA p = 0.21). C) A representative coronal brain section showing the contralateral axons from neurons co-transfected with GFP and DISC1ΔC. Line scans were used to measure the average signal intensity across the entire cortex and were normalized by the signal intensity found just above corpus colossum. The line scans were binned and averaged within each condition D) Summary plots showing no significant difference in the amount of axonal arborization between control and DISC1ΔC conditions. E) For statistical analysis data each condition was binned by anatomical layer. No significant difference was observed within each layer between each condition (control (n = 7) vs. DISC1ΔC (n = 6); layer 2/3 1.48±0.10 vs. 1.46±0.06 p = 0.81; layer 4 1.17±0.05 vs. 1.09±0.08 p = 0.41; layer 5 1.19±0.03 vs. 1.12±0.02 p = 0.09; layer 6 1.04±0.02 vs. 0.94±0.05 p = 0.08) F) A representative image of contralateral axons co-expressing GFP, synaptophysin-RFP (syp-RFP) and DISC1ΔC. Arrowheads show syp-RFP positive puncta that colocalize with GFP positive axonal varicosities. G) Group data showing no significant difference in the number of syp-RFP puncta per length of axon (control (n = 17) vs. DISC1ΔC (n = 12); p = 0.34). (TIF) [file pone.0034053.s003.tif]

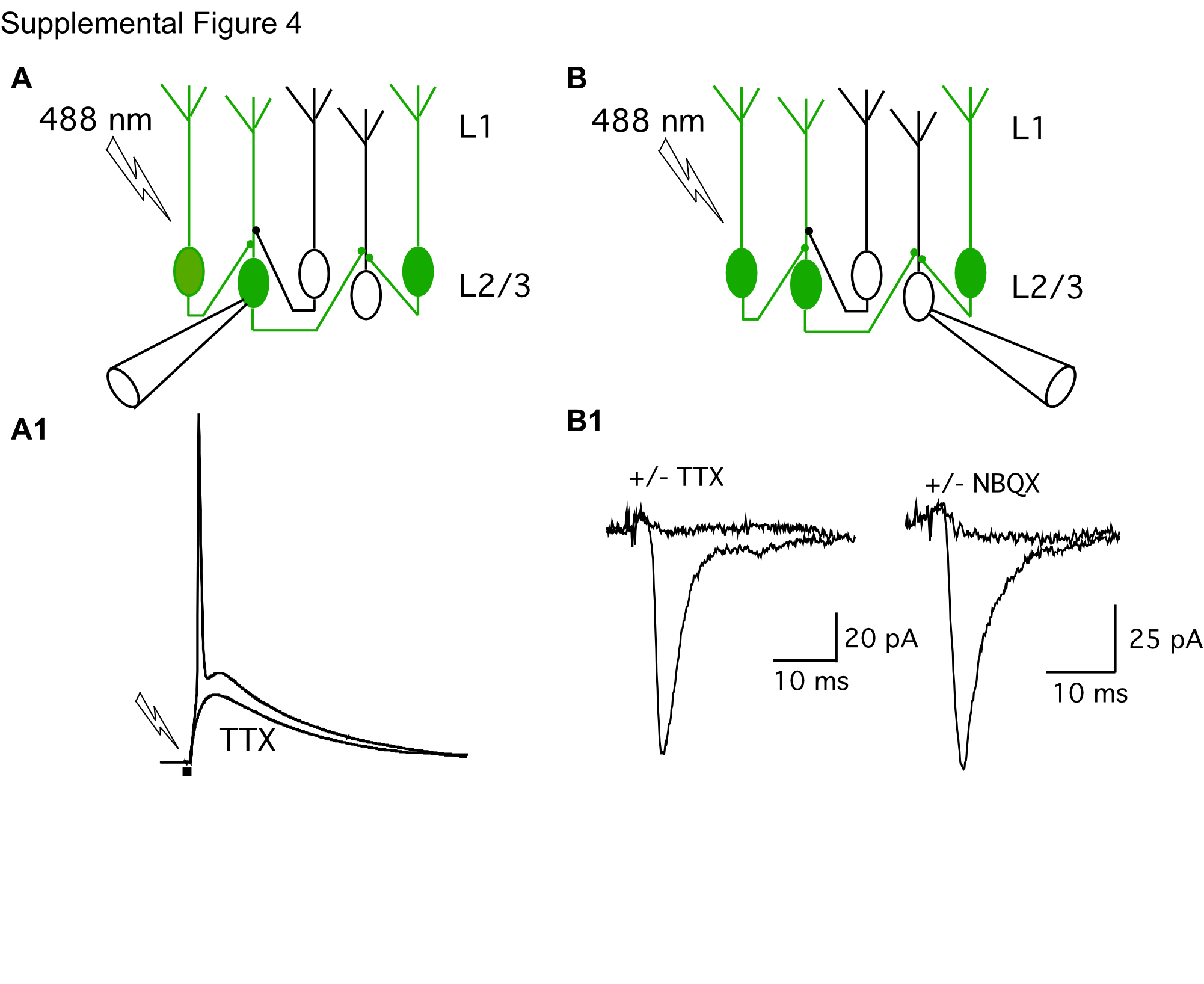

Supplement: Figure S4 — Activation of ChR2 with a 2 ms pulse of blue light evokes glutamatergic synaptic transmission. In utero electroporation on E15–E16 results in transfection of approximately 20% of layer 2/3 neurons in the targeted cortical area. Measuring light-activated synaptic transmission is amendable in this circuit because of the high level of recurrent connections between layer 2/3 neurons. A) Schematic depicting a recording in a layer 2/3 neuron transfected with ChR2 (green cell). (A1) A 2 ms pulse of blue light (473 nM; ∼1 mW) generates a single action potential in a control neuron that is blocked by TTX application. B) Schematic depicting a recording from an untransfected neuron (open cell) surrounded by layer 2/3 neurons transfected with ChR2. B1) A 2 ms pulse of blue light stimulates surrounding ChR2-positive neurons to fire action potentials and results in an EPSC in the untransfected neuron that is completely blocked by TTX (1 µM, 94.4±4.8% block of control response, n = 13, p<0.005) or the AMPA receptor antagonist NBQX (10 µM, 96.5±0.8% block of control response, n = 11, p<0.0005). All EPSC recordings performed in the presence of the GABAa antagonist (gabazine, 5 µM). (TIF) [file pone.0034053.s004.tif]

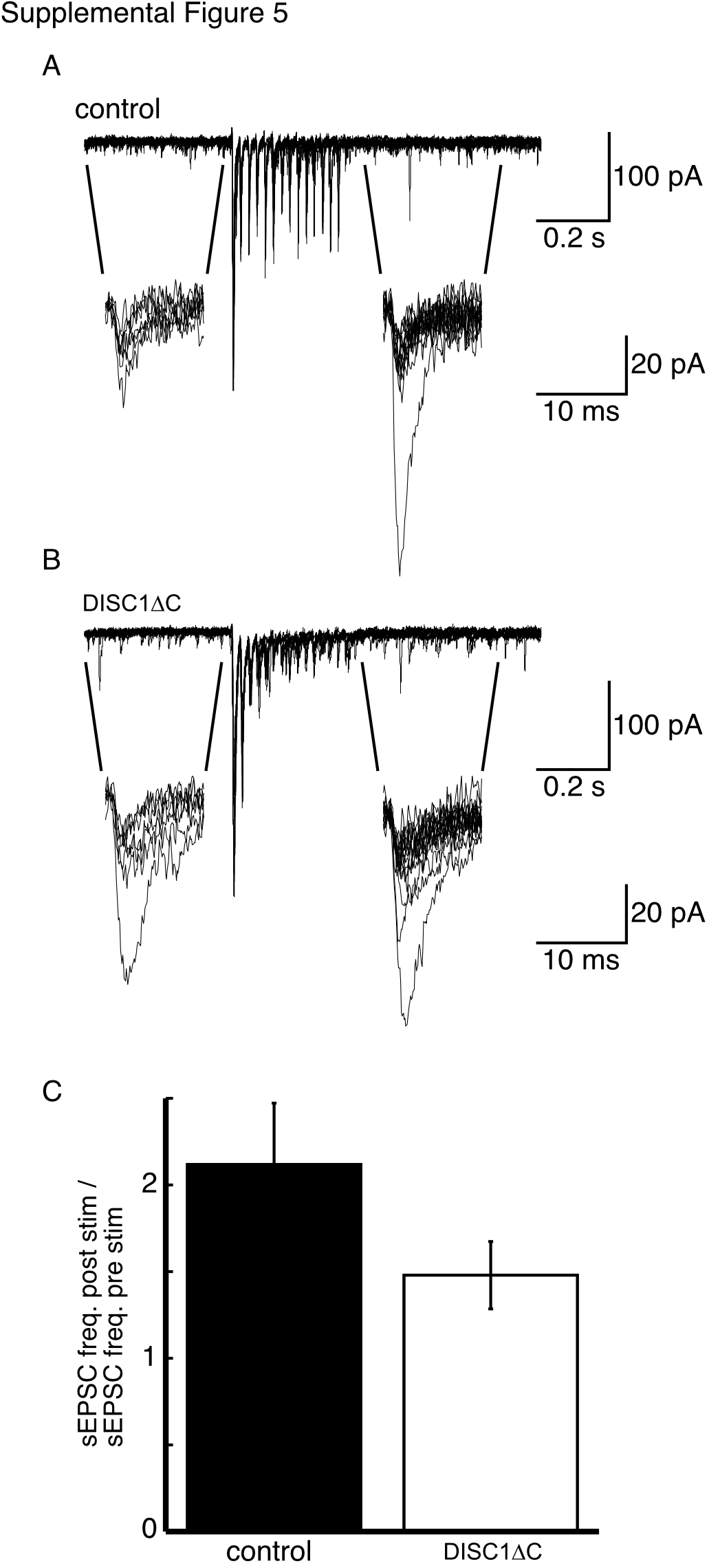

Supplement: Figure S5 — Presynaptic expression of DISC1ΔC does not alter asynchronous release. A) A representative train of EPSCs recorded from an untransfected neuron and evoked from presynaptic neurons expressing ChR2 and dsRed (control). Ten consecutive traces are overlaid. EPSCs were evoked with a train of 15 light pulses at 50 Hz. sEPSCs were collected 400 ms before and after the stimulus train (inset). B) A representative train of EPSCs recorded from an untransfected neuron and evoked from presynaptic neurons expressing ChR2 and DISC1ΔC. C) Summary data showing this stimulation protocol was effective in producing a significant asynchronous release for control recordings, as the frequency of sEPSCs is significantly enhanced following the stimulus train in control but not DISC1ΔC condition (control frequency before train 1.2±0.2 Hz vs. after train 2.6±0.6 Hz (n = 12); p<0.02 paired t-test; DISC1ΔC frequency before train 2.1±0.4 Hz vs. after train 2.6±0.4 Hz (n = 13); p = 0.2). However, the post stimulation/prior stimulation ratio of sEPSC frequency was not statistically different between control and DISC1ΔC terminals (control 2.12±0.36 (n = 12) vs. DISC1ΔC 1.48±0.19 (n = 13); p = 0.13), suggesting the DISC1ΔC–dependent slowing of EPSC kinetics is separate from asynchronous release. (TIF) [file pone.0034053.s005.tif]
